# A Strengths, Weaknesses, Opportunities, and Threats (SWOT) Analysis of ChatGPT Integration in Nursing Education: A Narrative Review

**DOI:** 10.7759/cureus.48643

**Published:** 2023-11-11

**Authors:** Ahmad A Abujaber, Alaa Abd-alrazaq, Ahmad R Al-Qudimat, Abdulqadir J Nashwan

**Affiliations:** 1 Department of Nursing, Hamad Medical Corporation, Doha, QAT; 2 AI Center for Precision Health, Weill Cornell Medicine-Qatar, Doha, QAT; 3 Department of Public Health, Qatar University, Doha, QAT; 4 Surgical Research Section, Department of Surgery, Hamad Medical Corporation, Doha, QAT

**Keywords:** swot analysis, nursing education, large language models, gpt-4, chatgpt

## Abstract

Amidst evolving healthcare demands, nursing education plays a pivotal role in preparing future nurses for complex challenges. Traditional approaches, however, must be revised to meet modern healthcare needs. The ChatGPT, an AI-based chatbot, has garnered significant attention due to its ability to personalize learning experiences, enhance virtual clinical simulations, and foster collaborative learning in nursing education. This review aims to thoroughly assess the potential impact of integrating ChatGPT into nursing education. The hypothesis is that valuable insights can be provided for stakeholders through a comprehensive SWOT analysis examining the strengths, weaknesses, opportunities, and threats associated with ChatGPT. This will enable informed decisions about its integration, prioritizing improved learning outcomes. A thorough narrative literature review was undertaken to provide a solid foundation for the SWOT analysis. The materials included scholarly articles and reports, which ensure the study's credibility and allow for a holistic and unbiased assessment. The analysis identified accessibility, consistency, adaptability, cost-effectiveness, and staying up-to-date as crucial factors influencing the strengths, weaknesses, opportunities, and threats associated with ChatGPT integration in nursing education. These themes provided a framework to understand the potential risks and benefits of integrating ChatGPT into nursing education. This review highlights the importance of responsible and effective use of ChatGPT in nursing education and the need for collaboration among educators, policymakers, and AI developers. Addressing the identified challenges and leveraging the strengths of ChatGPT can lead to improved learning outcomes and enriched educational experiences for students. The findings emphasize the importance of responsibly integrating ChatGPT in nursing education, balancing technological advancement with careful consideration of associated risks, to achieve optimal outcomes.

## Introduction and background

Integrating artificial intelligence (AI) technologies has shown great promise in revolutionizing teaching and learning processes across various educational contexts [1]. Within the field of nursing education, ChatGPT (Chat Generative Pre-Trained Transformer, a predictive language generation software program developed by OpenAI) has emerged as a noteworthy AI tool that deserves attention. As a generative AI chatbot (a software application designed to simulate human conversation, either written or spoken, by utilizing a combination of predefined scripts and AI technologies), ChatGPT offers versatility by providing personalized and interactive support to learners, thus significantly enhancing their educational experiences [2,3]. However, before fully embracing and implementing ChatGPT in nursing education, it is crucial to understand its benefits and thoroughly assess the associated risks. This comprehensive evaluation allows for the optimal utilization of its strengths and opportunities while effectively mitigating its weaknesses and threats.

Recognizing the potential transformative power of ChatGPT (referring to ChatGPT's capacity to revolutionize industries, behaviors, and tasks through its advanced conversational abilities, making interactions more efficient, personalized, and insightful across various domains), it becomes paramount to capitalize on its strengths and opportunities to ensure its effective integration into nursing education. ChatGPT can potentially be employed as a virtual tutor in nursing education, assisting students with queries and offering study materials [4]. Additionally, it could be used in simulation scenarios, helping nurses practice patient interactions and hone diagnostic reasoning without real-life consequences, thus enhancing learning experiences and promoting critical thinking. ChatGPT is proven effective at triaging high-acuity cases and could aid in identifying critical care needs, and with further medical training, its accuracy for other triage categories may improve [5]. Simultaneously, addressing the weaknesses and threats inherent in AI technology is vital to mitigating any potential negative consequences. By thoroughly analyzing ChatGPT's potential impact on nursing education, stakeholders can make informed choices that optimize the benefits and minimize the risks. This strategic approach enables the field of nursing education to leverage the advantages offered by ChatGPT while safeguarding against any potential challenges.

The primary objective of this review is to conduct a comprehensive SWOT (Strengths, Weaknesses, Opportunities, and Threats) analysis of ChatGPT's role in supporting nursing education. By examining its strengths, weaknesses, opportunities, and threats, this analysis aims to provide an in-depth understanding of the tool's potential benefits, limitations, and challenges within the nursing education context and to provide a well-rounded assessment that helps stakeholders make informed decisions.

The analysis is meticulously structured into four sections, corresponding to each component of the SWOT analysis tool: strengths, weaknesses, opportunities, and threats. The research team has conducted an exhaustive examination of previous literature within each section. This thorough analysis offers stakeholders an informative and comprehensive summary, facilitating the integration decision-making process.

Strengths and weaknesses in the SWOT analysis refer to the internal elements of an organization, business, or industry [6-8]. The strengths section delves into the advantages of AI-integrative chatbot tools, highlighting their potential benefits and positive impacts on nursing education. By thoroughly assessing these strengths, stakeholders gain a deeper understanding of these tools' value to the educational landscape. Conversely, the weaknesses section critically evaluates the limitations and challenges of AI-integrative chatbot tools in nursing education. This scrutiny gives stakeholders a realistic perspective, enabling them to anticipate and address potential obstacles during implementation.

Opportunities and threats in the SWOT analysis are an organization's external elements or environmental factors [6-8]. The opportunities section explores the potential avenues for growth and development that integrating AI-integrative chatbot tools presents in nursing education. It sheds light on innovative possibilities and ways to enhance teaching and learning experiences. Lastly, the threats section examines the external factors that may pose challenges or risks to the successful integration of AI-integrative chatbot tools in nursing education. By identifying these threats, stakeholders can proactively devise strategies to mitigate risks and ensure responsible and effective use of such tools.

The culmination of these four sections provides a comprehensive and informative summary derived from a thorough analysis of previous literature. This equips stakeholders with the knowledge and insights necessary to make informed decisions about the utilization of AI-integrative chatbot tools in nursing education. Ultimately, this study may help harness the potential of tools such as ChatGPT while ensuring optimal learning outcomes and ethical considerations in nursing education.

## Review

Methods

Literature Review Procedure

An exhaustive narrative literature review was conducted to ensure a comprehensive understanding and a solid foundation for the subsequent SWOT analysis. The following steps outline the methodology employed:

1. Search Strategy: Multiple databases, including PubMed, Scopus, and Google Scholar, were searched to identify relevant articles and studies with no time restrictions. Keywords and phrases such as “ChatGPT”, “Chatbot”, “Generative Pre-Trained Transformer”, “GPT”, and “Nursing Education” in conjunction with the specific topic under investigation were used to refine the search results.

2. Selection Criteria: Initial searches yielded 4,600 potential articles. These were then screened based on relevance to the study's focus. Any literature that did not offer significant insight into the subject matter or was outdated was excluded from the review.

3. Data Extraction: Relevant information and insights were extracted from the selected articles. This included key findings, theoretical concepts, and any potential factors that could contribute to the SWOT analysis.

4. Analysis and Synthesis: The information was critically analyzed once the data extraction was complete. Similar findings were grouped based on SWOT themes. The synthesis process helped in identifying the primary strengths, weaknesses, opportunities, and threats pertinent to the topic.

SWOT analysis

Strengths

Nursing education is a demanding field that requires constant adaptation and innovation to meet the needs of students and instructors alike [9]. ChatGPT technology has revolutionized nursing education delivery in recent years, offering a range of previously impossible advantages [10]. Through improved communication, access to a wealth of resources, and enhanced learning through personalized chatbot interactions, ChatGPT has become an indispensable tool for nursing education. This section further explores these advantages and highlights why ChatGPT is necessary for any nursing education program.

The importance of communication between instructors and students has been widely recognized in educational research. For example, a study by Corn et al. (2011) [11] emphasized the need for improved communication between instructors and students. The study found that communication is crucial in enhancing student learning experiences and academic achievement. Effective communication can help instructors understand their students' needs and tailor their teaching methods to meet those needs. Moreover, students who feel comfortable communicating with their instructors are more likely to seek help and engage more in learning [12]. Another study by Borup and Evmenova (2019) [13] suggests that instructors should employ various communication methods, including email, online discussion boards, and face-to-face interactions, to establish and maintain open lines of communication with their students. Instructors are also encouraged to provide timely feedback to students and to be approachable and responsive to their inquiries [13]. Furthermore, the study highlights the importance of communication in improving the quality of education and calls for instructors to prioritize communication as a fundamental aspect of their teaching practice. In this aspect, ChatGPT has the potential to facilitate seamless communication between nursing instructors and students by offering instant, clarifying information, bridging knowledge gaps, and providing a platform for interactive learning [14]. This promotes clearer understanding and continuous feedback and fosters a collaborative educational environment, enhancing the overall learning experience [15].

Nursing resources have become increasingly important as the nursing profession continues to evolve. These resources offer valuable information, tools, and support for nurses to enhance their knowledge and skills, ultimately improving patient outcomes. According to Salifu et al. (2019) [16], accessing a wealth of nursing resources is crucial for continuing education and professional development. These resources include online databases, journals, textbooks, and professional organizations. Online databases such as PubMed and CINAHL provide access to the latest research and evidence-based practice guidelines. Journals offer rich information on clinical practice, theory, and interdisciplinary collaboration. Textbooks provide in-depth knowledge on specific topics and serve as a foundation for nursing education. Professional organizations such as the American Nurses Association (ANA) and the National Association of Pediatric Nurse Practitioners (NAPNP) offer networking opportunities, continuing education courses, and advocacy for the nursing profession. Access to these resources benefits individual nurses and contributes to the advancement of the nursing profession. Nurses need access to these resources to stay current and informed, ultimately improving the quality of patient care. Integrating ChatGPT into the nursing education ecosystem has the potential to revolutionize access to key resources by simplifying information retrieval. Acting as a dynamic assistant, it directs users to pertinent resources and enhances real-time knowledge acquisition. Its capabilities offer instant insights, aid educators and students, and effectively bridge the gap between nurses and essential information [17].

Personalized learning has recently become popular in education [18]. One way to achieve this is through chatbots, which can provide students with individualized feedback, support, and guidance. According to a study by Kuhail et al. (2023) [19], personalized chatbot interactions can significantly enhance learning outcomes. The study found that students who interacted with a chatbot tailored to their individual needs and interests showed improved performance compared to those who received generic feedback. The personalized chatbot is adapted to the student's learning style, pace, and preferences, providing the necessary support to achieve their learning goals. The study also highlighted the importance of the design and development of the chatbot to ensure that it can provide accurate and relevant feedback to the student. This suggests that personalized chatbot interactions can revolutionize how we approach education, making it more individualized and effective.

The use of ChatGPT in nursing education offers numerous advantages, one of which is its ability to provide real-time responses and personalized feedback to nursing students [2]. This accessibility is crucial in the fast-paced and demanding world of healthcare, as it allows students to have immediate access to information and support. Students can ask questions and receive tailored responses that cater to their unique learning needs, leading to a more engaging and effective educational experience. Furthermore, the instant feedback provided by ChatGPT can enable students to quickly identify areas for improvement, fostering a continuous learning process that is essential in nursing education.

Another advantage of using ChatGPT in nursing education is its capacity to provide a wide range of resources and materials [20]. With the ever-evolving nature of medical knowledge and best practices, access to up-to-date information is crucial for educators and students. ChatGPT can compile and deliver relevant resources from various sources, including research articles, textbooks, case studies, and guidelines from healthcare organizations [21]. However, Sun and Hoelscher underscored that while ChatGPT offers revolutionary capabilities, it's not without challenges, particularly concerning integrity [20]. Educators must champion ethical use, equipping students for a technology-centric healthcare environment [22]. This enables nursing students to dive deeper into specific topics, enhancing their understanding of complex concepts and helping them stay informed about the latest advancements in their field.

Furthermore, ChatGPT offers a valuable tool for nursing educators by facilitating the creation of engaging and interactive learning experiences for students [23,24]. By incorporating ChatGPT into classroom activities, simulations, or discussion forums, educators can introduce an element of interactivity that captures students' interest and promotes active learning. For instance, ChatGPT can simulate realistic patient interactions or complex case scenarios, allowing students to practice their clinical reasoning and decision-making skills in a safe and controlled environment [25]. Moreover, by providing a platform for dynamic group discussions, ChatGPT fosters collaboration and critical thinking, essential nursing practice competencies [26-29].

In conclusion, ChatGPT offers numerous advantages for nursing education. Its advanced technology and innovative features make it a powerful tool for nursing students and educators. With its ability to generate relevant and accurate responses to questions, provide personalized feedback, and even simulate patient interactions, ChatGPT is an asset to anyone looking to improve their nursing education. Using ChatGPT, nursing students can better understand complex concepts, enhance their critical thinking skills, and become more effective healthcare professionals. With all these benefits and more, ChatGPT is a valuable resource for anyone looking to excel in nursing.

Weaknesses

Limitation of knowledge: Currently, the knowledge of ChatGPT is limited to the period before 2021 based on the datasets used in ChatGPT training [30]. Several studies have reported limitations in ChatGPT's knowledge, including wrong citations and examples of citations of non-existent sources [31,32]. Furthermore, two case studies advised against using ChatGPT, citing concerns about limited knowledge, scientific inaccuracy, and the inability to analyze results [33,34]. Therefore, it is essential to carefully review content generated by ChatGPT before using it. Several concerns and challenges might arise with the use of ChatGPT in nursing education, including the inability to identify information bias or mistakes, privacy concerns, decreased critical thinking, and creativity.

Lack of consistency: ChatGPT showed improved language comprehension levels, especially negative expressions and antonyms [32,35,36]. Its ability to maintain consistency in negation showed noteworthy advancements compared to its previous version. Despite the general notion of ChatGPT's exceptional performance, it needs to meet expectations regarding overall consistency [37]. It frequently alters decisions when presented with a paraphrased version of an input text. This indicates the self-contradictory nature of the application. Additionally, ChatGPT may make different decisions when changing the order of input sentences. Considering how natural symmetry is in human thinking, symmetric consistency violations significantly harm ChatGPT's reliability [37]. One way to mitigate this issue is by providing ChatGPT with different and appropriate text input and more ChatGPT training, as the specific construction of the prompt may result in diverse responses [38]. ChatGPT's lack of consistency impacts nursing education, as it may only sometimes be able to identify errors or inaccuracies in the content it generates.

Lack of reliability: ChatGPT has been known to generate content that may need to be more original, excessively detailed, or consistent, potentially causing additional challenges, especially in healthcare [32,35, 39-41]. An important concept required for healthcare decision-making and research is understanding the complexity of psychological and biological systems that ChatGPT cannot achieve [42,43]. ChatGPT cannot be relied upon as a reliable source in the meantime [44]. ChatGPT's unreliability may affect nursing education by providing inappropriate information about nursing interventions, illnesses, and medication management.

Misinformation and algorithmic bias: According to several studies, there are concerns about potential bias in ChatGPT's training datasets. This bias may restrict its abilities and lead to factual inaccuracies [38,45]. Additionally, it is essential to consider security concerns and potential cyberattacks regarding the propagation of misinformation through LLM [45]. When it comes to nursing education and academic writing, it is crucial to consider inaccurate data, potential abuse concerns such as factual inaccuracies, ethical considerations, and the spread of misinformation [39,46,47]. A common criticism of using ChatGPT for academic writing is that it often produces content that is considered superficial or inaccurate [31,39,48-50]. Ethical concerns such as the potential for bias from the training data set and the risk of plagiarism were often raised, characterizing ChatGPT as a "black box" technology [32,39-41,49-55]. So, the ability of ChatGPT to generate misinformation content seems scientifically plausible [56]. The generation of inaccurate information can lead to negative consequences in health settings [57-60]. On the other hand, algorithmic bias is associated with imprecise data, and the resulting output relies entirely on sophisticated algorithms with no human involvement, posing risks in nursing education and medical education [57]. Borji extensively discusses the limitations of using ChatGPT; this includes various factors such as the creation of imprecise information, the potential for partiality and prejudice, the absence of clarity and dependability, apprehensions regarding cybersecurity, ethical issues, and broader impacts on society [61]. ChatGPT's misinformation and bias impact nursing education through no human monitoring, possible bias, and affect patient safety.

Lack of human emotion: Computer science and linguistics aim to create AI capable of executing tasks that typically necessitate human intelligence [62]. This includes emotions and emotional intelligence [63]. However, one of the problems with using ChatGPT applications in healthcare is that the app needs help understanding people's emotional reasoning [54,57]. As a result, there are concerns that human emotions may be underestimated. It is, therefore, important to emphasize the role of health professionals in addressing the potential psychological, economic, and social consequences that may arise from the use of such tools in a health setting [41-43].

Opportunities

With the advancement of technology, the integration of AI is becoming increasingly common across various industries. Healthcare is exploring AI's use in clinical and academic education. Generative AI models such as ChatGPT offer numerous opportunities for the nursing, clinical and academic education sectors. The integration of this technology can enhance student learning by providing a readily accessible source of information, personalized learning experiences, and the latest developments in the nursing field. As AI continues to evolve, it will undoubtedly play an increasingly critical role in nursing education, providing students with a powerful tool to enhance their learning and ultimately improve patient outcomes. The optimistic scholars on the AI chatbot discussed several opportunities where generative AI chatbots could help address fundamental issues in the education system and revolutionize education.

Early identification of learning needs: It is widely known that systematic assessment of learning needs is of utmost importance. It is necessary to develop training curricula to facilitate learners' learning processes and provide the basis for continuing professional development [64,65]. Scholars have developed various tools and models to improve the effectiveness of needs assessment [65]. Even with the pace of change in the work environment and the increasing working demands, the early identification of emerging learning needs is exceptionally challenging. Early identification of learning needs is essential because it can help prevent learning difficulties from becoming more serious and ensure that students receive the support they need to succeed in school. First, generative AI chatbots may promptly provide personalized suggestions to support the learner's needs [66]. The dynamic interaction between the students and the chatbot and the personalized and adaptive feedback may serve as a means for the students and teachers to capture emerging learning needs, which can be used to design need-based learning materials for both classroom and clinical education.

The importance of systematically assessing learning needs cannot be overstated, as it is crucial for developing effective training curricula and facilitating learners' educational processes [64]. Scholars have developed various tools and models to enhance the effectiveness of needs assessment [65]. However, due to the rapid pace of change in the work environment and increasing demands, identifying emerging learning needs early on has become increasingly challenging. Early identification of learning needs is crucial, as it can prevent learning difficulties from becoming more serious and ensure that students receive the support they need to succeed in school [67].

Firat proposed using generative AI chatbots that promptly provide personalized suggestions to support the learner's needs [66]. This approach leverages the dynamic interaction between students and chatbots and personalized and adaptive feedback to capture emerging learning needs. These needs can then be used to design need-based learning materials for both classroom and practical education. By leveraging this technology in nursing education, nursing students and instructors can capture and address emerging learning needs, leading to improved educational outcomes.

Address the traditional education barriers: Scholars believe that the integration of AI chatbots into the education system has the potential to address three significant challenges to learning in classrooms: knowledge transfer, evaluation, and the illusion of explanatory depth. According to Mollick and Mollick [68], teachers could utilize AI chatbots to achieve the following objectives:

a) Facilitate knowledge transfer by providing students with diverse examples and explanations over time. This approach can help students reference previous knowledge when faced with new problems, enhancing their understanding of concepts.

b) Improve students' evaluation skills by training them to critically evaluate essays generated by AI chatbots. This exercise can help students develop the ability to assess the validity and reliability of information and arguments presented to them.

c) Address the illusion of explanatory depth by encouraging students to recognize and acknowledge gaps in their knowledge about a topic. Students can take a step back, reassess their understanding, and seek additional information to deepen their knowledge and comprehension.

Overall, addressing these barriers via the integration of AI chatbots into the nursing education system can improve nursing students' learning outcomes by providing them with personalized and engaging learning experiences and contribute to increasing nursing students' satisfaction [69].

Support self-directed learning: With the rapid pace of advancements in the digital world, the demand for individuals who can navigate the ever-changing landscape is increasing. These individuals are highly motivated to engage in self-directed learning [70]. In today's rapidly changing world, professionals are compelled to personalize their education to meet their specific needs, and self-directed learning is more likely to achieve this goal [71]. Self-directed learning has gained popularity in nursing education [72], with scholars finding that it leads to improved learning outcomes, increased satisfaction with competency and clinical practice learning among nursing students, and the development of independence, professional autonomy, increased choice, and motivation when blended with appropriate coaching [72,73].

Firat [66] proposed that generative AI chatbots can enhance self-directed learning in six ways. First, they offer personalized learning by providing tailored and interactive help to the self-learners. Second, chatbots offer real-time feedback and guidance to assist students in staying focused on their tasks. Third, chatbots are more accessible as they can be used on various platforms, including smartphones. Fourth, learning becomes more convenient and flexible since students can study using chatbots at their own pace and terms. Fifth, chatbots can enhance open educational resources by helping self-directed learners find and utilize various online learning tools and materials. Lastly, chatbots can be used for self-assessment and reflection, enabling learners to reflect on their progress and identify areas that require further support.

Support personalized education: Similarly, several researchers have emphasized the importance of providing personalized self-learning support [74]. Personalized learning is customized instruction that considers individual needs and goals [75]. Studies have shown that personalized learning is a practical approach that can increase motivation, engagement, and understanding [76], particularly in clinical education, where it has been shown to improve student outcomes [77]. Generative chatbots can offer personalized learning experiences by being adaptive based on students' progress and providing personalized, targeted feedback [67]. A scoping review conducted by Buchanan et al. maintained that AI chatbots could give customized feedback to nursing students and improve their clinical skills and learning outcomes if combined with a strong foundation in nursing education [78]. Additionally, the implementation of mobile chatbot learning among nursing students in Taiwan was found to encourage self-directed learning, promote learning achievements, and enhance their self-efficacy [79].

Support virtual clinical simulation: Virtual simulations have been used in clinical education for several years. Several scholars discussed that AI-based virtual avatar applications, including virtual tutor chatbots, may influence the delivery of nursing education in academic settings as educators use them as teaching tools to simulate interactive clinical scenarios, increase students' comprehension of specific nursing concepts, and improve their reasoning and decision-making skills [80,81]. Using chatbots in virtual simulations can enhance the learning experience by creating realistic virtual scenarios that allow students to practice their clinical skills in a safe environment [82,83].

Support collaborative learning: collaborative (AKA cooperative) learning is a teaching approach involving students actively engaging with one another to achieve a shared learning objective [84]. This instructional method can take various forms, such as group projects, discussions, and peer-to-peer teaching, and emphasizes the development of teamwork, communication, and critical thinking skills among students [85,86]. As discussed by Zhang and Cui, the clinical environment is becoming increasingly complex; hence, nursing education must respond promptly by reforming current course curricula and seeking innovative teaching strategies to prepare better nursing graduates to meet larger demands [85]. Collaborative learning effectively enhances nursing students' critical thinking, social interactions, learner autonomy, and learning success [86]. AI chatbots can facilitate cooperative education by providing a platform for learners or professionals to share ideas, solve problems, and work together towards shared objectives [85,87].

Threats

Academic Dishonesty

Using generative AI tools like GPT-4 and ChatGPT to answer short-answer and multiple-choice exam questions can potentially be exploited for cheating. These tools can also produce medical essays that are difficult to distinguish from those written by humans, which could lead to increased plagiarism. While many tools (e.g., GPTZero, Originality.AI, and Turnitin AI Writing Detector) have been developed to detect AI-generated text, students may still be able to make their AI-generated essays undetected by such tools. According to a study, the addition of a single word ("amazing") to an AI-generated text decreased the level of detection of the text being generated by AI from 99% to 24% [24]. Although this is just one example, it still highlights apprehensions regarding such tools' effectiveness in detecting and preventing plagiarism.

Overreliance

As previously noted, recent generative AI tools, such as GPT-4, can potentially present inaccurate information more convincingly and believably [88]. This can result in users trusting these tools excessively, thereby increasing the risk of overreliance. Consequently, using generative AI tools may impede the development of essential skills in nursing students, including critical thinking, problem-solving, and communication. In other words, the convenience of these tools for providing answers could reduce students' motivation to investigate and arrive at their conclusions or solutions independently. This raises a crucial question about how generative AI tools can enhance, rather than diminish, students' critical thinking and problem-solving abilities.

Inequity in Access

The availability of generative AI tools may exacerbate the disparity between students, as only some have equal access. While these tools can communicate in several languages besides English, their proficiency in each language depends on the quality and quantity of available training data in each language [88]. Consequently, individuals who need to improve in English are more likely to use these tools. Additionally, generative AI tools may be less accessible to those lacking technological skills, who lack access to technology such as computers and the internet, cannot afford subscription fees (e.g., the USD 20/month for GPT-4), or have disabilities like blindness or motor impairment.

Privacy

When students and educators interact with generative AI tools, they may inadvertently disclose personal information such as their name, email, phone number, prompts, uploaded images, and generated images. OpenAI acknowledges that it may use this information for various purposes, such as analyzing, maintaining, and improving its services, conducting research, preventing fraud, criminal activity, or misusing its services, and complying with legal obligations and processes [89]. Furthermore, OpenAI may share this personal data with third parties without further notice or consent from the users [89]. Italy's data protection group recently halted access to such tools to investigate data collection and usage practices that align with the General Data Protection Regulation (GDPR) [90]. Additionally, using these tools during clinical rotations for patient care (e.g., SOAP note generation) may lead to accidental patient privacy breaches. The safety and security of student and patient data should be central to discussions on the curriculum.

Copyrights

Generative AI tools may be trained on copyrighted materials such as books, scientific articles, and images. This may lead to creating text that bears similarities to or directly copies content protected by copyright, potentially impacting downstream uses. This situation raises concerns about utilizing content generated by such tools (e.g., educational materials, presentations, course syllabi, quizzes, and scientific papers) without appropriate acknowledgment and authorization from the copyright holder. Discussions regarding authorship rights for articles written using generative AI tools are ongoing. Some publishers and journals do not accept listing such tools as Co-Author, while others do. As this is an evolving area, it raises questions about how students and educators should acknowledge using these tools while complying with professional and regulatory expectations.

Table 1 summarizes the findings of the SWOT analysis.

**Table 1:** Summary of the SWOT Analysis

Category	Strengths	Weaknesses	Opportunities	Threats
Accessibility	Provides 24/7 access to educational resources and support	Not yet available on all smartphones	Enhances remote education for students in rural or remote areas	Technical issues
Consistency	Delivers standardized and unbiased information	The level of consistency is directly influenced by the precision of the provided prompts.	Facilitates global collaboration between nursing educators and students	Resistance to change from traditional nursing educators and institutions
Adaptability	Customizable to individual student needs, adapting to their learning pace and style	Limited human interaction hinders the development of empathy and soft skills	Creates blended learning environments by integrating with traditional nursing education	Overreliance on AI potentially hinders critical thinking and problem-solving skills
Cost-effective	Reduces the need for additional human resources, lowering educational costs	Cannot provide hands-on experience or evaluate practical skills	By the projected timeline, the subscription fees are anticipated to become more economical, especially for nursing students.	Regulatory and legal hurdles regarding data privacy and intellectual property
Updating	Continuously updated with the latest nursing research, clinical guidelines, and best practices	Vulnerable to data breaches and may misunderstand complex or nuanced questions	Supports lifelong learning and continuing education for practicing nurses	Technological limitations may result in outdated or incomplete information
Others		Incomplete knowledge base due to limitations in training data	Can be integrated into simulation training programs to improve clinical decision-making	Ethical concerns, such as potential biases in AI-generated responses

Limitations

Despite the exhaustive nature of this SWOT analysis, some limitations inherent to the SWOT framework still exist. It is largely subjective, relying heavily on the interpretive abilities of those conducting the analysis, which can lead to potential bias or an overemphasis on certain aspects of the study [91]. The SWOT analysis also presents a static view, which might not accurately reflect AI's dynamic and evolving nature in education, particularly in the rapidly changing field of nursing education. Furthermore, it doesn't inherently provide a strategy for managing the identified strengths, weaknesses, opportunities, and threats. For example, while this study identifies the potential benefits of ChatGPT in terms of accessibility and adaptability, it does not suggest concrete strategies for optimizing these strengths or addressing the weaknesses and threats, such as dependency on technology or privacy concerns.

Recommendations

To facilitate the responsible integration of ChatGPT in nursing education, several recommendations can be made for educators, nursing students, and policymakers. Educators may need to embrace ChatGPT as a supplementary tool and receive adequate training to effectively incorporate it into their teaching practices. This allows them to explore innovative approaches, such as designing interactive modules, virtual simulations, and collaborative learning environments that leverage the capabilities of ChatGPT. On the other hand, nursing students need to be encouraged to actively engage with ChatGPT, utilizing it as a resource for personalized support and additional learning materials while maintaining a balanced approach that promotes critical thinking and independent learning. Policymakers have a crucial role in shaping the integration of ChatGPT in nursing education. They must collaborate with educators and AI developers to establish clear guidelines and best practices that ensure responsible use, protect student privacy, address ethical considerations, and ensure compliance with copyright and academic integrity standards. Additionally, policymakers need to advocate for equitable access to ChatGPT to bridge the digital divide and promote inclusivity in nursing education. By implementing these recommendations, educators, nursing students, and policymakers can navigate the integration of ChatGPT effectively, maximizing its benefits while upholding ethical standards and ensuring the best possible educational outcomes for nursing students.

## Conclusions

In conclusion, this comprehensive SWOT analysis highlights the potential of ChatGPT to support nursing education while acknowledging the ethical and practical challenges it presents. Stakeholders in nursing education can make informed decisions by leveraging the identified strengths and opportunities while proactively addressing the weaknesses and threats. Collaboration among educators, policymakers, and AI developers is crucial to ensuring the ethical and effective use of ChatGPT. Integrating ChatGPT as a supplementary tool allows nursing educators to provide personalized support, interactive modules, and virtual simulations to enhance student understanding and engagement. Emphasizing responsible use, protecting student privacy, and addressing copyright and academic integrity concerns are vital in developing guidelines and best practices for successful integration. Striking the right balance between technology and human involvement in nursing education allows for the transformative potential of ChatGPT while upholding ethical standards and ensuring optimal educational outcomes. Future research should focus on investigating the long-term impact of ChatGPT on learning outcomes, addressing ethical considerations, and developing guidelines that foster critical thinking and problem-solving skills in nursing education. These research directions will contribute to the ongoing development and implementation of ChatGPT, providing valuable insights for educators, policymakers, and AI developers to optimize its benefits and address potential challenges.
